# Recent advances in the development of alkyne metathesis catalysts

**DOI:** 10.3762/bjoc.7.12

**Published:** 2011-01-18

**Authors:** Xian Wu, Matthias Tamm

**Affiliations:** 1 Institut für Anorganische und Analytische Chemie, Technische Universität Braunschweig, Hagenring 30, 38106 Braunschweig, Germany

**Keywords:** alkynes, homogeneous catalysis, metathesis, molybdenum, tungsten

## Abstract

The number of well-defined molybdenum and tungsten alkylidyne complexes that are able to catalyze alkyne metathesis reactions efficiently has been significantly expanded in recent years.The latest developments in this field featuring highly active imidazolin-2-iminato- and silanolate–alkylidyne complexes are outlined in this review.

## Review

### Introduction

C–C bond formation is one of the most important types of reaction in organic synthesis. Transformations employing organometallic compounds as catalysts have achieved a significant role because of their advantages such as simplicity (fewer reaction steps) and efficiency (higher yields) in comparison with traditional synthetic strategies. Nowadays, a plethora of methods is known, which can be used for the formation of C–C single and double bonds, whereas simple ways to create C–C triple bonds are less common, despite the importance and ubiquity of C–C triple bonds in research areas such as natural product synthesis and advanced material science [1].

**Scheme 1:**
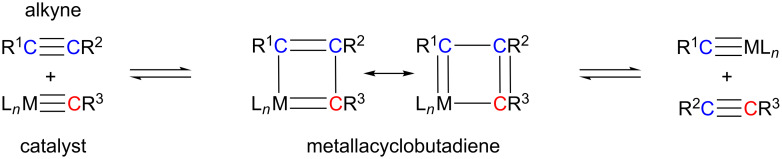
Alkyne metathesis based on the Katz mechanism.

**Scheme 2:**
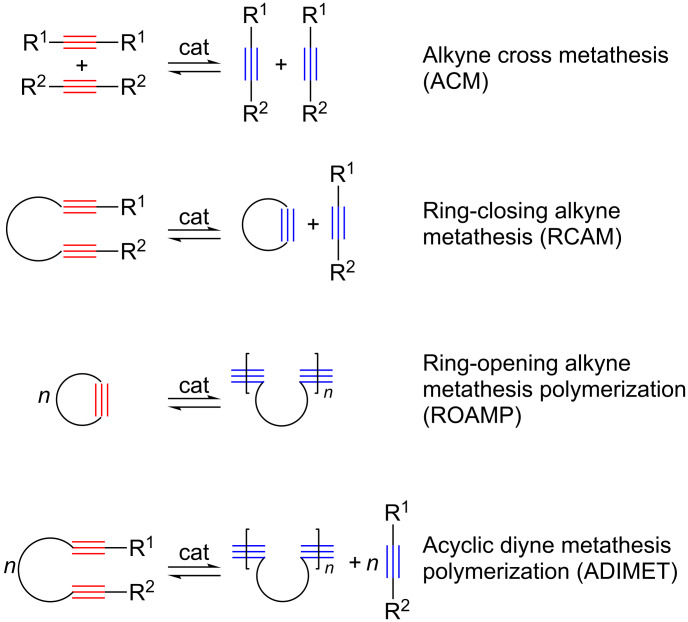
Reaction patterns of alkyne metathesis.

Alkyne metathesis, which deals with the breaking and making of C–C triple bonds, has only relatively recently become part of the tool box of organic and polymer chemists for the preparation of their target molecules [2–11]. Catalyzed by organotransition metal complexes, this reaction type creates new C–C triple bonds very simply via the Katz mechanism (Scheme 1) [12], based on which a series of different reaction types such as alkyne cross metathesis (ACM), ring-closing alkyne metathesis (RCAM), ring-opening alkyne metathesis polymerization (ROAMP) and acyclic diyne metathesis polymerization (ADIMET) are known (Scheme 2).

In contrast to olefin metathesis, the number of catalysts for alkyne metathesis is far more limited. The first catalyst for alkyne metathesis was a heterogeneous system based on WO_3_/silica, which was first reported by Pennella, Banks and Bailey in 1968 [13], while the first homogeneous system, which consisted of [Mo(CO)_6_] and resorcinol [14], was discovered by Mortreux and Blanchard in 1974. Since then, great efforts have been made to develop highly efficient alkyne metathesis catalysts and this has led to three major systems which have dominated this area, i.e., the Mortreux system, the Schrock system and the Cummins–Fürstner–Moore system. Only recently, two novel systems, which exhibit highly promising catalytic performance in alkyne metathesis, were successfully introduced: 1. A modified Schrock system containing imidazolin-2-iminato ligands that was developed by our group; 2. silanolate-supported complexes such as molybdenum nitride and alkylidyne complexes with Ph_3_SiO ligands developed by Fürstner and tungsten alkylidyne complexes with (*t*-BuO)_3_SiO ligands introduced by us. Since there are already several reviews available that cover research progress up to 2006 [2–11], this article will focus on the two novel catalyst systems, which were established over the last four years (2007–10), commencing with a brief introduction to the established systems that have already been widely used by synthetic chemists.

### Traditional catalyst systems

#### Mortreux system

First reported in 1974, the Mortreux system consists of two components: [Mo(CO)_6_] and phenol or derivatives thereof [14–19]. During the last decades, this system was intensively studied and its performance was significantly improved. However, some drawbacks including the requirement of high reaction temperatures and low functional group tolerance greatly limit its applicability. Moreover, the catalytic mechanism and the active species involved remain unknown, preventing a further rational catalyst design. Nevertheless, because of the commercial availability and high stability of the pre-catalysts as well as the simplicity of operation, this classical system is still widely used by chemists [20–28].

#### Schrock system

Schrock-type catalysts are high oxidation state molybdenum or tungsten alkylidyne complexes which form metallacyclobutadienes (the key intermediate in the Katz mechanism) upon treatment with internal alkynes. Among these, the tungsten neopentylidyne complex [Me_3_C≡CW(OCMe_3_)_3_] is the most widely used species and is reliably synthesized in several steps from commercially available WCl_6_. Accordingly, numerous applications of this catalyst have been reported, which usually requires elevated reaction temperatures and relatively high catalyst loadings [29–35].

#### Cummins–Fürstner–Moore system

**Scheme 3:**
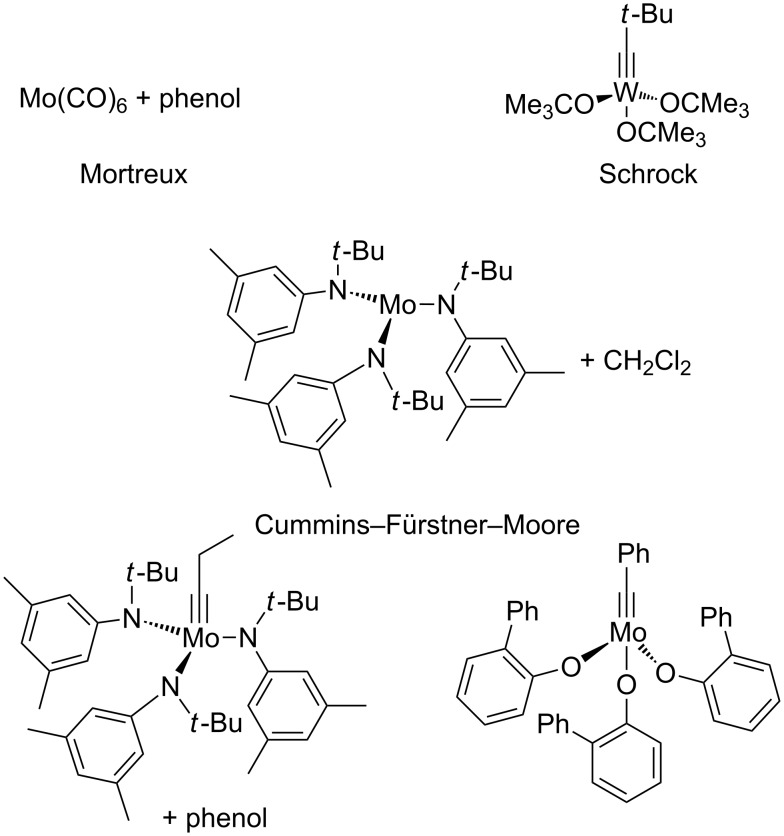
Typical examples from traditional catalyst systems.

Cummins introduced triamido molybdenum(III) complexes of the type [Mo{NR(Ar)}_3_] in the mid 1990s, which are able to cleave the N–N triple bond in the dinitrogen molecule [36–38]. Based on this discovery, Fürstner developed a catalyst system that is formed upon treatment of [Mo{N(*t*-Bu)Ar}_3_] with dichloromethane to give the methylidyne complex [HC≡Mo{N(*t*-Bu)Ar}_3_] and the chloro complex [ClMo{N(*t*-Bu)Ar}_3_] [39]. Although the detailed reaction mechanism has not been fully uncovered, the latter complex is, somewhat counterintuitively, considered to be the active species. Similarly, Moore was able to isolate molybdenum alkylidyne complexes such as [EtC≡Mo{N(*t*-Bu)Ar}_3_], which are able to catalyze alkyne metathesis reactions efficiently, albeit only after treatment with phenol derivatives or by capture on silica [40–46]. The reaction with phenolic compounds presumably leads to partial or complete cleavage of the Mo–N bonds to produce catalytically active phenolate complexes. In agreement with this assumption, Cummins was able to report the synthesis of well-defined molybdenum benzylidyne complexes from the molybdaziridine [Mo(H)(η^2^-Me_2_CNAr){N(*i*-Pr}Ar)] and could demonstrate that these systems are efficient initiators for alkyne metathesis even at ambient temperature and low catalyst loadings [47]. Scheme 3 shows some typical examples of the three traditional catalyst systems.

### Novel catalyst systems

#### Imidazolin-2-iminato tungsten and molybdenum alkylidyne complexes

**Scheme 4:**
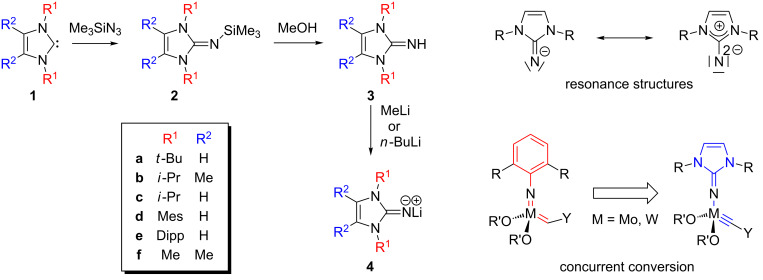
Ligand synthesis and catalyst design.

Imidazolin-2-iminato ligands, which are isolobal to phosphoraneimides (R_3_PN^−^) and cyclopentadienides (C_5_R_5_^−^) [48–52], can be described by the resonance structures shown in Scheme 4, indicating that the ability of the imidazolium ring to stabilize a positive charge affords highly basic ligands with a strong electron-donating capacity towards early transition metals or metals in a higher oxidation state [53–55]. In recent years, our group has significantly expanded the use of these 2σ,4π-electron donor ligands in organometallic chemistry and homogeneous catalysis [56–67]. Their synthesis starts from *N*-heterocyclic carbenes **1** which react with trimethylsilyl azide to afford 2-trimethylsilyliminoimidazolines **2**. After treatment with methanol, the corresponding imidazolin-2-imines **3** can be conveniently isolated [60]. Deprotonation by alkyl lithium reagents leads to imidazolin-2-iminato lithium compounds **4**, which serve as ligand transfer reagents during the catalyst preparation (Scheme 4).

The idea to use imidazolin-2-iminato ligands for the modification of Schrock-type alkylidyne complexes is based on the consideration that they can be regarded as monoanionic analogues of dinegative imido ligands, which are present in some of the most active olefin metathesis catalysts, i. e., Schrock–Hoveyda-type tungsten and molybdenum imido-alkylidene complexes [10]. We presumed that substitution of the imido ligands by imidazolin-2-iminato ligands and concurrent conversion of the metal–carbon double bond into a triple bond would afford metal alkylidyne species with a well-preserved structural and electronic integrity, and therefore with potentially undiminished catalytic activity (Scheme 4). Thus, the resulting new complexes should then be highly active alkyne metathesis catalysts.

**Scheme 5:**
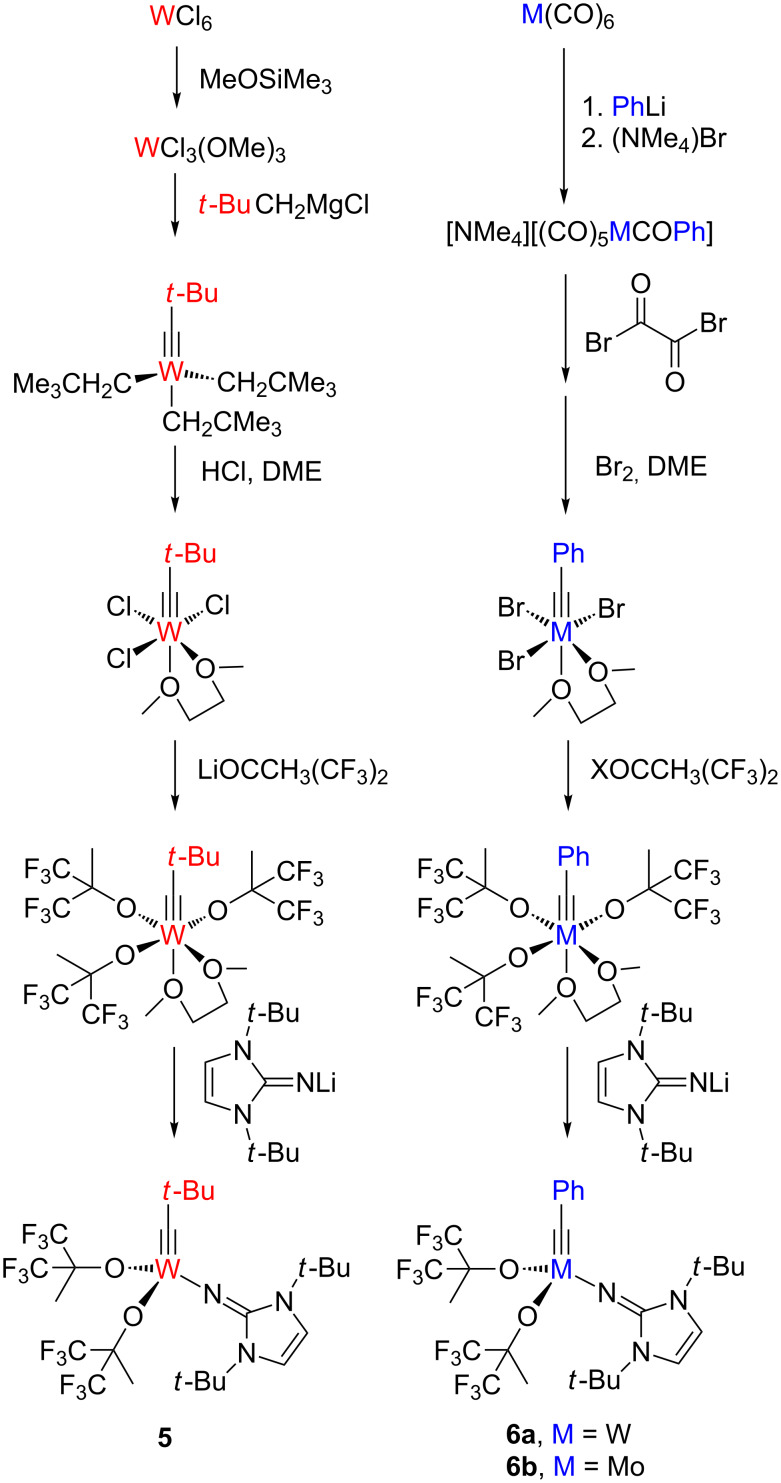
Catalysts synthesis using high- and low-oxidation-state routes (for **6a**, X = Li or K; for **6b**, X = K).

In order to verify this design strategy, high oxidation state tungsten and molybdenum alkylidyne complexes bearing imidazolin-2-iminato ligands (**5** and **6**) were synthesized by two different routes. The low-oxidation-state route (on the right-hand side in Scheme 5) starting from metal hexacarbonyl has advantages such as higher atom economy, easier operation and suitability for both tungsten and molybdenum [68–70] in comparison with the high-oxidation-state route (on the left- hand side in Scheme 5) starting from tungsten hexachloride [71–73]. The use of partially fluorinated alkoxides such as hexafluoro-*tert*-butoxide, OCCH_3_(CF_3_)_2_, proved to be essential for creating active catalysts [74], indicating that successful catalyst design in this system relies on establishing a push-pull situation in a similar fashion present in Schrock–Hoveyda olefin metathesis catalysts (Scheme 4) [10] and also in an isolobal rhenium(VII) imido-alkylidyne complex [Re(NAr)(C*t*-Bu)(OR^F^)] (Ar = 2,6-diisopropylphenyl, R^F^ = CCH_3_(CF_3_)_2_), which is able to metathesize aliphatic alkynes [75]. In contrast, however, anionic molybdenum imido-alkylidyne complexes such as [Mo(NAr)(C*t*-Bu)(OR^F^)]^−^ do not promote alkyne metathesis, since the more electron-rich nature of the alkylidyne anion may disfavor alkyne binding [76].

**Figure 1:**
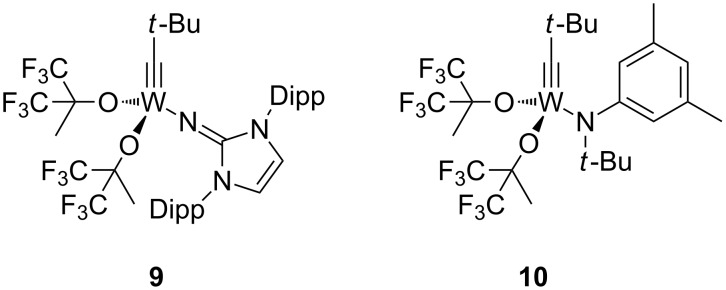
Alkylidyne complexes **9** and **10**.

The catalysts **5** and **6** were proved to catalyze various alkyne metathesis reactions including ACM, RCAM and ROAMP. In addition, the isolation and structural characterization of a metallacyclobutadiene complex from the reaction of **5** with an excess of 3-hexyne confirmed that the [2 + 2]-cycloaddition (Katz) mechanism is operative [73–74]. The prototype **5** of our new catalyst system was used for the ACM of 1-phenylpropyne (**7**) and was shown to be significantly more active than the classic Schrock alkylidyne complex [Me_3_CC≡W(O*t*-Bu)_3_] at both ambient and elevated temperatures [73–74]. Its performance was also compared with those of two other catalysts **9** and **10** bearing Im^Dipp^N and N(*t*-Bu)Ar ligands, respectively (Table 1, Figure 1). The results show that **5** is significantly more active than **9** and **10**, whereas **10** is more active than **9**. This is supported by DFT calculations for the metathesis of 2-butyne as the model reaction, which reveal that the activation barrier for the three catalysts follows the order **9** > **10** > **5**.

**Table 1:** ACM of **7** using **5**, **9** and **10** as catalysts.

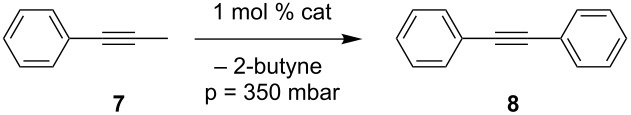				

cat	temp (°C)	*t* (min)	solvent	yield (%)

**5**	ambient	50	hexane	100
**5**	80	40	toluene	100
**9**	ambient	50	hexane	2
**9**	80	40	toluene	6
**10**	ambient	50	hexane	28
**10**	80	40	toluene	89

ACM reactions with more complex substrates bearing different functional groups were studied in the presence of **6a** and **6b** as catalysts [70]. In the ACM of the 3-pentynyl ether **11**, tungsten and molybdenum benzylidyne complexes **6a** and **6b** were used as catalysts, both showing excellent activities under the same vacuum-driven reaction conditions (Table 2). In our hands, however, the tungsten system appeared to be a superior and more reliable catalyst system than its molybdenum congener, which was also supported by DFT calculations. Similar results were also found for the ACM of the 3-pentynyl benzoic esters **13** bearing a selection of functional groups in the 4-position of the phenyl ring (Table 2). With the tungsten catalyst **6a**, excellent yields were obtained for X = Cl, OMe and SMe, whereas only 17% of **14d** could be obtained for X = NO_2_. Increasing the catalyst loading to 2 mol % gave a higher conversion (33%), and we have obtained similar results for other substrates. For instance, ACM of **13e** (X = NMe_2_) was hardly successful in the presence of 2 mol % of the catalyst, whereas **14e** was isolated in 90% yield with a catalyst loading of 5 mol %. Further detailed studies are required to fully explain this ostensibly odd behavior.

**Table 2:** ACM of **11** and **13** using **6a** and **6b** as catalysts.

substrate	product	catalyst	yield (%)^a^	

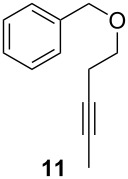	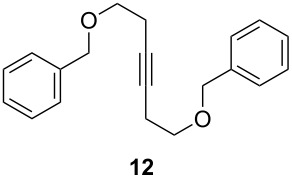	**6a** **6b**		98 97
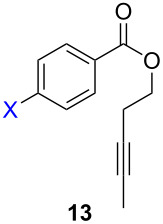	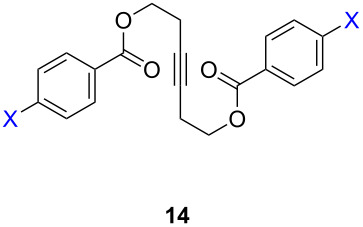	**6a** **6b**	**a** X = H **a** X = H	98 97
		**6a** **6a** **6a** **6a** **6a**	**a** X = H **b** X = Cl **c** X = OMe **d** X = NO_2_ **e** X = NMe_2_	98 98 94 17 90^b^

^a^1 mol % catalyst, toluene, rt, 1 h, 200 mbar. ^b^5 mol % catalyst, toluene, rt, 2 h, 200 mbar, unpublished results.

Catalyst **5** was used in the RCAM of 6,15-dioxaeicosa-2,18-diyne (**15**) and *o*-, *m*- and *p*-bis(3-pentynyloxymethyl)benzenes **17** (Table 3). While the cyclic product **16** was obtained from **15** in high yield (95%), different selectivities toward the formation of monomeric [10]cyclophanes **18** and [10.10]cyclophanes **19** depending on the substitution pattern were observed [77]: The monomeric cycloalkyne **18b** and the dimeric cyclodiyne **19c** were exclusively formed from the *m*- and *p*-isomer **17b** and **17c**, respectively, whereas ring-closure of the *o*-isomer **17a** gave a mixture of both **18a** and **19a**. This observation is in agreement with DFT calculations suggesting that reversible ring-opening and ring-closing metathesis (RORCM) leads to an equilibrium between monomeric and dimeric products and their ratios are determined by their relative stabilities [77].

**Table 3:** RCAM of **15** and **17** using **5** as catalyst.

substrate	product	yield (%)^a^	

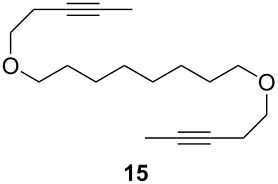	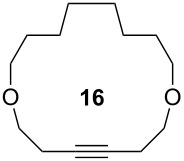		95
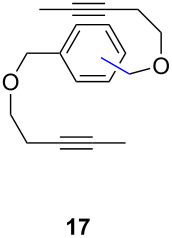	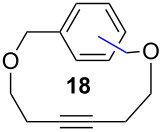	**a** *ortho* **b** *meta* **c** *para*	24 100 0
	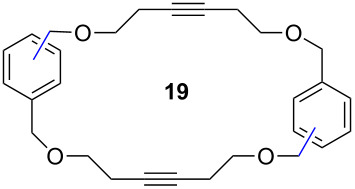	**a** *ortho* **b** *meta* **c** *para*	76 0 100

^a^2 mol % catalyst **5**, hexane, rt, 2 h, 350 mbar.

The catalytic performance of **6a** and **6b** in RCAM was demonstrated for the substrates *m*-bis(3-pentynyloxymethyl)benzene (**17b**) and bis(3-pentynyl)phthalate (**20**) (Table 4). The results showed that the tungsten benzylidyne complex can catalyze both reactions with high efficiency, whereas the molybdenum counterpart had a significantly lower activity, in agreement with a theoretically predicted higher activation barrier for the Mo system [70].

**Table 4:** RCAM of **17b** and **20** using **6a** and **6b** as catalysts.

substrate	product	catalyst	yield (%)^a^

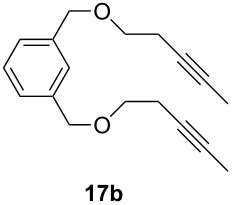	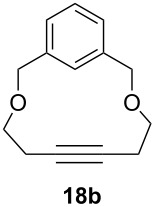	**6a** **6b**	86 47
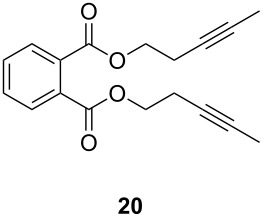	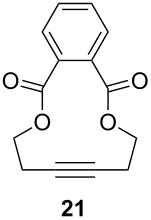	**6a** **6b**	98 20

^a^2 mol % catalyst, 80 mL toluene, rt, 2 h, 300 mbar.

The ROAMP of cyclooctyne (**22**) was performed using **5** and **6a** as catalysts (Table 5) [78]. According to gel permeation chromatography (GPC) analysis, polymer parameters such as the molecular weight (*M*_n_ and *M*_w_) and the polydispersity index (PDI) depend on the catalyst and substrate concentration, and the reaction medium. Besides polymer formation, cyclooligomers were also detected by GPC and mass spectrometry. As shown in Table 5, both catalysts **5** and **6a** catalyzed the ring-opening metathesis polymerization efficiently. It is also found that high yields of polymer were obtained when the reactions were performed on neat substrate, whereas lower substrate concentration increases the formation of cyclooligomers. This observation can be well explained by the Jacobson–Stockmayer theory of ring-chain equilibria [79].

**Table 5:** Ring-opening alkyne metathesis polymerization of cyclooctyne using **5** and **6a** as catalysts.

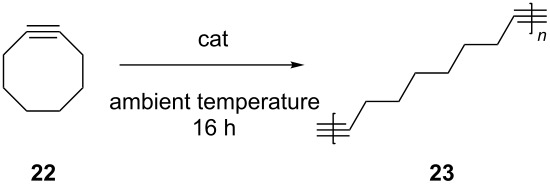							

cat	mol %	solvent	*c*_sub_ (mol/L)	*M_n_* (g/mol)	*M_w_* (g/mol)	PDI	polymer yield (%)

**5**	1	-	neat	33000	46800	1.4	70
**6a**	1	-	neat	26400	41300	1.6	80
**6a**	5	-	neat	9960	23200	2.3	95
**6a**	5	toluene	0.03	82000	100000	1.2	7
**6a**	5	toluene	0.02	-^a^	- ^a^	- ^a^	0
**6a**	5	*n*-hexane	0.02	- ^a^	- ^a^	- ^a^	0

^a^only cyclic oligomers were obtained.

#### Molybdenum nitride and alkylidyne complexes with silanolate ligands

**Scheme 6:**
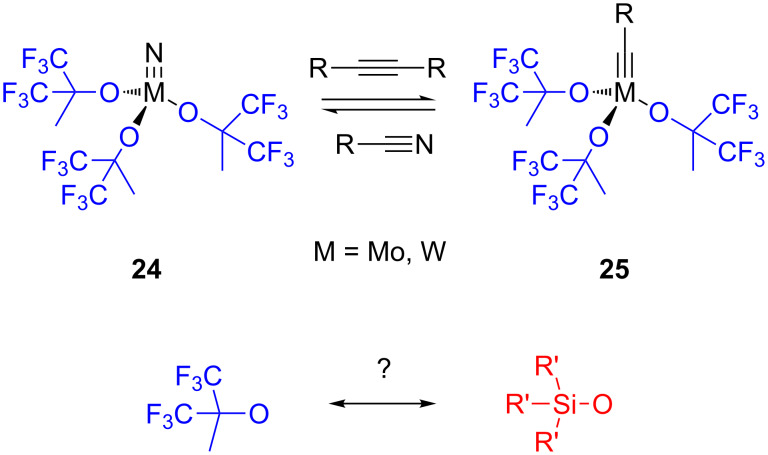
Design strategy of Fürstner’s new system.

**Scheme 7:**
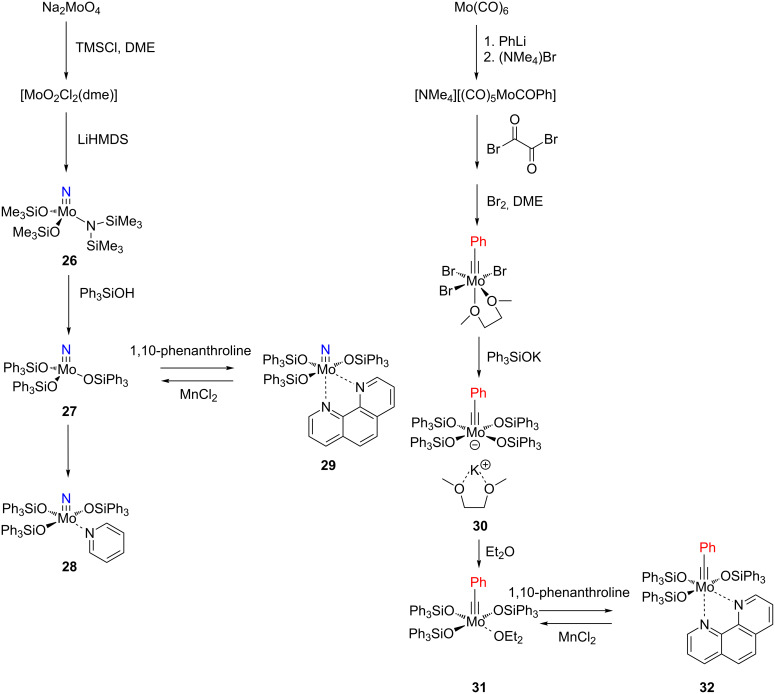
Synthetic routes of Fürstner’s new catalysts.

Fürstner recently established a different design strategy for the development of novel alkyne metathesis catalysts. Inspired both by a report of Johnson and co-workers, who found that molybdenum and tungsten nitride complexes **24** with fluorinated alkoxide ligands react with alkynes to generate the corresponding metal alkylidynes **25** in situ (Scheme 6) [80–81], and by the work of Chiu et al. on the preparation of a silanolate-supported molybdenum-nitride complex [82], Fürstner’s group introduced a novel user-friendly catalyst system for alkyne metathesis by employing triphenylsilanol (Ph_3_SiOH) [83–84]. Two synthetic routes were developed, which are shown in Scheme 7. The one on the left-hand side starting from Na_2_MoO_4_ leads to molybdenum nitride pre-catalysts, while the one on the right-hand side starting from [Mo(CO)_6_] directly affords molybdenum alkylidyne complexes. This procedure resembles the low-oxidation-state route presented in Scheme 5.

The catalytic activities of the complexes **26**, **28**, **29**, **31**, **32** in ACM and RCAM were studied for a variety of substrates. In their initial publication [83], catalytic reactions were performed using **26**/Ph_3_SiOH and **28** as catalysts. Although satisfactory to good yields were achieved, all reactions required elevated reaction temperatures (≥ 80 °C) and, in most cases, high catalyst loadings (up to 20%). However, the results were greatly improved for the 1,10-phenanthroline (phen) systems **29**/MnCl_2_ and **32**/MnCl_2_ – MnCl_2_ is added to remove the phen-ligand by precipitation of MnCl_2_•phen – and for the diethyl ether (Et_2_O) complex **31** by addition of molecular sieves (MS 5 Å) to adsorb the 2-butyne formed during the metathesis reaction [84]. This method constitutes a significant advance, since it allows all reactions to be run in a closed system at ambient pressure. Accordingly, only the latter, improved results will be presented here, and Table 6 and Table 7 summarize the results for ACM and RCAM with the pre-catalysts **29**/MnCl_2_, **31** and **32**/MnCl_2_ [84].

**Table 6:** ACM of **33** using **29**/MnCl_2_, **31** and **32**/MnCl_2_ as catalysts.

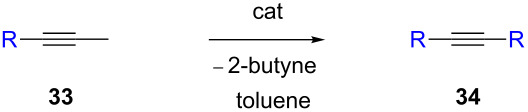				

**33**	R-	catalyst and yield (%)		
		**29**/MnCl_2_^a^	**31**^b^	**32**/MnCl_2_^c^

**a**^f^	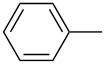	99	99	99
**b**	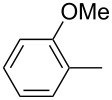	96	97	97
**c**	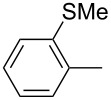	87	98^d^	96^d^
**d**	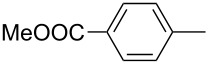	72^e^	95	97
**e**	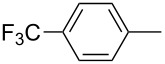	94	93	95
**f**	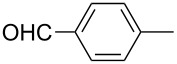	no reaction	no reaction	no reaction
**g**	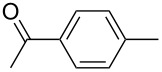	<40^e^	84	84
**h**	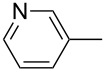	76^e^	90^d^	88^d^
**i**	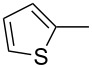	86	88	87
**j**	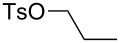	95	92	92
**k**	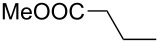	85	89	81
**l**	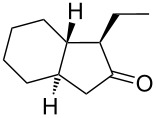		92	88
**m**	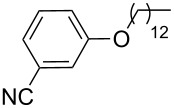	81	87	89

^a^**29** (10 mol %), activated by MnCl_2_ (10 mol %) at 80 °C, molecular sieve, 80 °C. ^b^**31** (2 mol %), molecular sieve, ambient temperature. ^c^**32** (5 mol %), activated by MnCl_2_ (5 mol %) at 80 °C, molecular sieve, ambient temperature. ^d^50 °C. ^e^100 °C. ^f ^**33a** and **34a** are the same as **7** and **8**, respectively.

**Table 7:** RCAM of **35** using **29**/MnCl_2_, **31** and **32**/MnCl_2_ as catalysts.

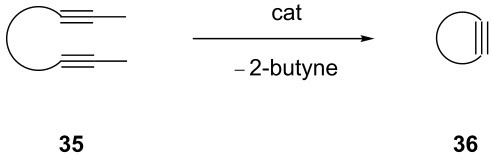				

**35**		catalyst and yield (%)		
		**29**/MnCl_2_^a^	**31**^b^	**32**/MnCl_2_^c^

**a**	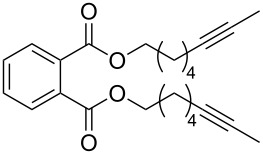	70	97	94
**b**	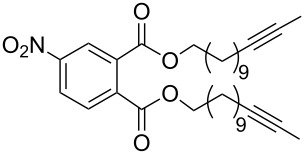		85	
**c**	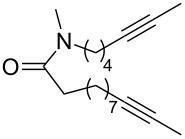	67	72	
**d**	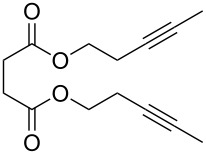	91	73	78
**e**	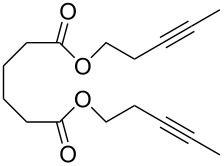	85	92	90

^a^**29** (10 mol %), activated by MnCl_2_ (10 mol %) at 80 °C, without molecular sieve, 80 °C. ^b^**31** (2 mol %), molecular sieve, ambient temperature. ^c^**32** (5 mol %), activated by MnCl_2_ (5 mol %) at 80 °C, molecular sieve, ambient temperature.

The air-stable nitride complex **29** performs satisfactorily in the presence of MnCl_2_ and MS 5 Å, however, its stability comes at the expense of higher catalyst loadings (10 mol %) and elevated reaction temperatures. In contrast, the phenanthroline–alkylidyne system **32** requires higher temperatures (80 °C) only for the activation with MnCl_2_, whereas the metathesis reaction can be carried out at ambient temperature. Noteworthy, it is the Et_2_O complex **31** that sets a new standard in alkyne metathesis, despite its reduced robustness in comparison with **32**. Like **29** and **32**, **31** – in combination with MS 5 Å – shows an excellent functional group tolerance together with a significantly enhanced catalytic performance even at lower catalyst concentrations and temperatures than indicated in Table 6 and Table 7 [84]. In addition, this catalyst was employed for the synthesis of various bioactive natural products and also for the total synthesis of natural occurring macrolactides [85–86], confirming and highlighting the strong potential of alkyne metathesis as a tool in organic synthetic methodology [9].

**Scheme 8:**
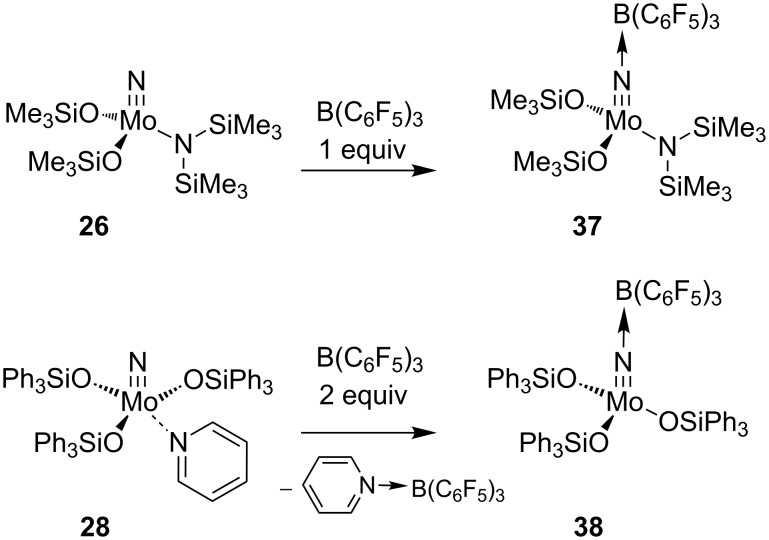
Lewis acid addition of **26** and **28**.

In a very recent report, Finke and Moore reported on the Lewis acid activation of the molybdenum nitrides **26** and **28**, which afforded the pre-catalysts **37** and **38** upon addition of one or two equivalents of B(C_6_F_5_)_3_, respectively (Scheme 8) [87]. While the adduct **38** is found to be active in alkyne metathesis, the complex **37** requires additional activation by treatment with the electron-poor phenol 2-(F_3_C)C_6_H_4_OH to facilitate the formation of a catalytically active molybdenum alkylidyne species. The latter system was tested for the metathesis of several phenylalkynes, and yields up to 64% were obtained by application of relatively forcing reaction conditions (10 mol % nitride, 20 mol % borane, 30 mol % phenol, *T* = 90 °C). Nevertheless, the rate of metathesis is enhanced in comparison with the performance of the borane-free complexes, and these results might therefore pave the way for the development of alkyne metathesis catalysts based on transition metal nitrides.

#### Silanolate-supported tungsten alkylidyne complexes

**Scheme 9:**
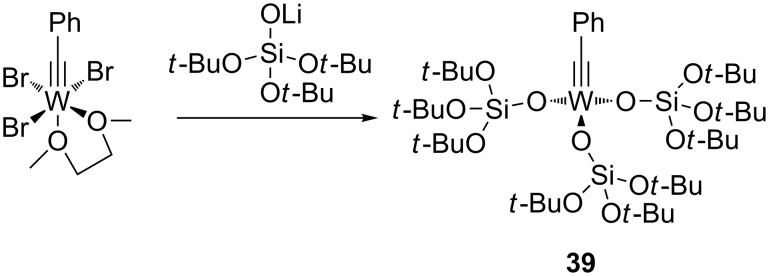
Preparation of the silanolate–alkylidyne tungsten complex **39**.

The suitability of silanolates as suitable ancillary ligands for the development of alkyne metathesis catalysts is further confirmed by our independent synthesis of the tungsten benzylidyne complex **39** (Scheme 9), which can be isolated in high yield as a yellow crystalline solid from the reaction of the tribromide [PhC≡WBr_3_(dme)] (dme = 1,2-dimethoxyethane) with the lithium salt of the silanol (*t*-BuO)_3_SiOH [88]. Since this silanol can be regarded as a mimic for silica surfaces [89–94], **39** might be regarded as a homogeneous model for silica-supported alkylidyne complexes [45–46,91–94]. Compound **39** exhibits excellent catalytic behavior in a number of ACM and RCAM reactions [88] (Table 8 and Table 9), and in analogy to Fürstner’s report [84], our studies also indicate that the addition of MS 5 Å does further improve the activity and the ease of applicability of this catalyst system.

**Table 8:** ACM using **39** as catalyst.

substrate	product	yield (%)^a^		yield (%)^b^	

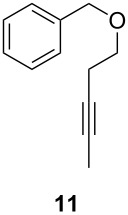	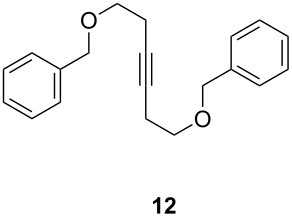	85		95	
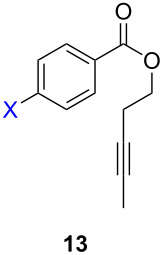	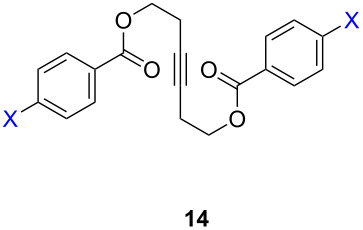	**a** X = H **b** X = Cl **c** X = OMe **d** X = SMe	97 92 96 94^c^	**a** X = H **b** X = Cl **c** X = OMe **d** X = SMe	99 97 97 99

^a^0.5 mmol substrate, 2 mol % catalyst **39**, 8 mL toluene, rt, 1 h, 200 mbar. ^b^0.5 mmol substrate, 1 mol % catalyst **39**, 2 mL toluene, rt, 1 h, 500 mg, molecular sieve. ^c^0.5 mmol, substrate, 5 mol % catalyst **39**, 8 mL toluene, rt, 1 h, 200 mbar.

**Table 9:** RCAM using **39** as catalyst.

substrate	product	yield (%)^a^	yield (%)^b^

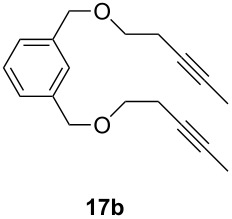	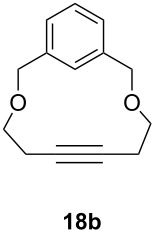	80	95
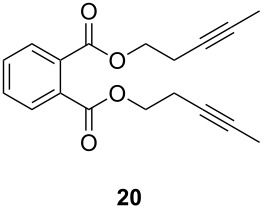	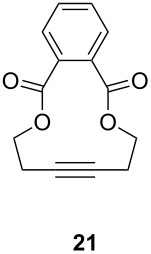	92	97
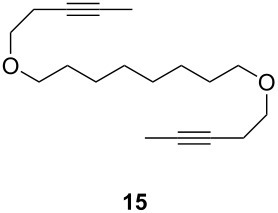	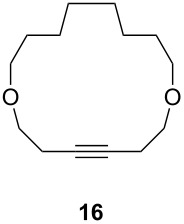	72	95
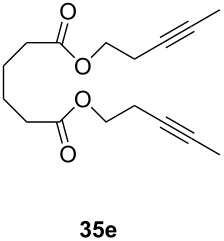	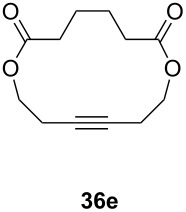	73	99

^a^0.36 mmol substrate, 2 mol % catalyst **39**, 80 mL toluene, 80 °C, 2 h. ^b^0.48 mmol substrate, 2 mol % catalyst **39**, 24 mL toluene, rt, 2 h, 1 g molecular sieve.

## Conclusion

“Although alkyne metathesis may never reach the breadth of alkene metathesis because of a smaller substrate base” [84], the recent additions to the comparatively small family of alkyne metathesis catalysts – imidazolin-2-iminato- and silanolate-supported molybdenum and tungsten alkylidyne complexes – should certainly help to boost the recognition of alkyne metathesis and to overcome the prevalence of olefin metathesis. The synthetic protocols developed for the synthesis of these new (pre-) catalysts allow for fine-tuning of their steric and electronic properties in order to further optimize their stability and catalytic performance and to modulate their structure according to the requirements of specific applications and substrate classes. However, the development in alkyne metathesis has yet to overcome one major obstacle, and that is the impracticability of employing terminal alkynes as substrates, since these tend to form polymers [95] and were also shown to degrade Schrock alkylidynes by formation of deprotonated, inactive metallacyclobutadienes [96]. Hence, future efforts should also re-address this issue, e. g., by adjusting the properties of the metallacyclobutadiene key intermediates [97] in order to prevent their degeneration and therefore ineffectiveness in undergoing the Katz [2 + 2]cycloaddition/cycloreversion mechanism (Scheme 1).

## References

[1] Diederich F, Stang P J, Tykwinski R R (2005). Acetylene Chemistry: Chemistry, Biology and Material Science.

[2] Zhang W, Moore J S (2007). Adv Synth Catal.

[3] Schrock R R, Czekelius C (2007). Adv Synth Catal.

[4] Van de Weghe P, Bisseret P, Blanchard N, Eustache J (2006). J Organomet Chem.

[5] Mortreux A, Coutelier O (2006). J Mol Catal A: Chem.

[6] Schrock R R (2005). Chem Commun.

[7] Bunz U H F (2005). Science.

[8] Schrock R R (2002). Chem Rev.

[9] Fürstner A, Davis P W (2005). Chem Commun.

[10] Schrock R R, Hoveyda A H (2003). Angew Chem, Int Ed.

[11] Fürstner A, Grubbs R (2003). Alkyne Metathesis. Handbook of Metathesis.

[12] Katz T J, McGinnis J (1975). J Am Chem Soc.

[13] Pennella F, Banks R L, Bailey G C (1968). Chem Commun (London).

[14] Mortreux A, Blanchard M (1974). J Chem Soc, Chem Commun.

[15] Mortreux A, Dy N, Blanchard M (1975). J Mol Catal.

[16] Mortreux A, Petit F, Blanchard M (1978). Tetrahedron Lett.

[17] Bencheick A, Petit M, Mortreux A, Petit F (1982). J Mol Catal.

[18] Mortreux A, Delgrange J C, Blanchard M, Lubochinsky B (1977). J Mol Catal.

[19] Mortreux A, Petit F, Blanchard M (1980). J Mol Catal.

[20] Kaneta N, Hikichi K, Asaka S-i, Uemura M, Mori M (1995). Chem Lett.

[21] Zhang W, Moore J S (2006). Angew Chem, Int Ed.

[22] Zhao D, Moore J S (2003). Chem Commun.

[23] Brizius G, Pschirer N G, Steffen W, Stitzer K, zur Loye H C, Bunz U H F (2000). J Am Chem Soc.

[24] Ge P H, Fu W, Herrmann W A, Herdtweck E, Campana C, Adams R D, Bunz U H F (2000). Angew Chem, Int Ed.

[25] Höger S (2005). Angew Chem, Int Ed.

[26] Miljanic O S, Vollhardt K P C, Whitener G D (2003). Synlett.

[27] Johnson C A, Lu Y, Haley M M (2007). Org Lett.

[28] Fürstner A, Guth O, Rumbo A, Seidel G (1999). J Am Chem Soc.

[29] Sancho J, Schrock R R (1982). J Mol Catal.

[30] Schrock R R, Clark D N, Sancho J, Wengrovius J H, Rocklage S M, Pederson S F (1982). Organometallics.

[31] Wengrovius J H, Sancho J, Schrock R R (1981). J Am Chem Soc.

[32] Fürstner A, Seidel G (1998). Angew Chem, Int Ed.

[33] Grela K, Ignatowska J (2002). Org Lett.

[34] Song D, Blond G, Fürstner A (2003). Tetrahedron.

[35] Fürstner A, Müller G (2000). J Organomet Chem.

[36] Laplaza C E, Odom A L, Davis M W, Cummins C C, Protasiewicz J D (1995). J Am Chem Soc.

[37] Laplaza C E, Cummins C C (1995). Science.

[38] Laplaza C E, Johnson A R, Cummins C C (1996). J Am Chem Soc.

[39] Fürstner A, Mathes C, Lehmann C W (1999). J Am Chem Soc.

[40] Zhang W, Kraft S, Moore J S (2003). Chem Commun.

[41] Zhang W, Kraft S, Moore J S (2004). J Am Chem Soc.

[42] Zhang W, Moore J S (2004). J Am Chem Soc.

[43] Zhang W, Moore J S (2005). J Am Chem Soc.

[44] Zhang W, Brombosz S M, Mendoza J L, Moore J S (2005). J Org Chem.

[45] Weissmann H, Plunkett K N, Moore J S (2006). Angew Chem, Int Ed.

[46] Cho H M, Weissmann H, Moore J S (2008). J Org Chem.

[47] Blackwell J M, Figueroa J S, Stephens F H, Cummins C C (2003). Organometallics.

[48] Dehnicke K, Greiner A (2003). Angew Chem, Int Ed.

[49] Dehnicke K, Krieger M, Massa W (1999). Coord Chem Rev.

[50] Dehnicke K, Weller F (1997). Coord Chem Rev.

[51] Dehnicke K, Strähle J (1989). Polyhedron.

[52] Diefenbach A, Bickelhaupt F M (1999). Z Anorg Allg Chem.

[53] Kuhn N, Göhner M, Grathwohl M, Wiethoff J, Frenking G, Chen Y (2003). Z Anorg Allg Chem.

[54] Kuhn N, Fawzi R, Steinmann M, Wiethoff J (1997). Z Anorg Allg Chem.

[55] Kuhn N, Fawzi R, Steinmann M, Wiethoff J, Bläser D, Boese R (1995). Z Naturforsch.

[56] Tamm M, Randoll S, Bannenberg T, Herdtweck E (2004). Chem Commun.

[57] Tamm M, Beer S, Herdtweck E (2004). Z Naturforsch.

[58] Tamm M, Randoll S, Herdtweck E, Kleigrewe N, Kehr G, Erker G, Rieger B (2006). Dalton Trans.

[59] Petrovic D, Tamm M, Herdtweck E (2006). Acta Crystallogr.

[60] Tamm M, Petrovic D, Randoll S, Beer S, Bannenberg T, Jones P G, Grunenberg J (2007). Org Biomol Chem.

[61] Panda T K, Randoll S, Hrib C G, Jones P G, Bannenberg T, Tamm M (2007). Chem Commun.

[62] Stelzig S H, Tamm M, Waymouth R M (2008). J Polym Sci, Part A: Polym Chem.

[63] Panda T K, Trambitas A G, Bannenberg T, Hrib C G, Randoll S, Jones P G, Tamm M (2009). Inorg Chem.

[64] Trambitas A G, Panda T K, Jenter J, Roesky P W, Daniliuc C, Hrib C G, Jones P G, Tamm M (2010). Inorg Chem.

[65] Tamm M, Trambitas A G, Hrib C G, Jones P G (2010). Terrae Rarae.

[66] Panda T K, Hrib C G, Jones P G, Tamm M (2010). J Organomet Chem.

[67] Trambitas A G, Panda T K, Tamm M (2010). Z Anorg Allg Chem.

[68] Mayr A, McDermott G A (1986). J Am Chem Soc.

[69] McDermott G A, Dorries A M, Mayr A (1987). Organometallics.

[70] Haberlag B, Wu X, Brandhorst K, Grunenberg J, Daniliuc C G, Jones P G, Tamm M (2010). Chem–Eur J.

[71] Schrock R R, Sancho J, Pederson S F (1989). Inorg Synth.

[72] Freudenberger J H, Schrock R R, Churchill M R, Rheingold A L, Ziller J W (1984). Organometallics.

[73] Beer S, Hrib C G, Jones P G, Brandhorst K, Grunenberg J, Tamm M (2007). Angew Chem, Int Ed.

[74] Beer S, Brandhorst K, Hrib C G, Wu X, Haberlag B, Grunenberg J, Jones P G, Tamm M (2009). Organometallics.

[75] Schrock R R, Weinstock I A, Horton A D, Liu A H, Schofield M H (1988). J Am Chem Soc.

[76] Tonzetich Z J, Schrock R R, Müller P (2006). Organometallics.

[77] Beer S, Brandhorst K, Grunenberg J, Hrib C G, Jones P G, Tamm M (2008). Org Lett.

[78] Lysenko S, Haberlag B, Wu X, Tamm M (2010). Macromol Symp.

[79] Monfette S, Fogg D E (2009). Chem Rev.

[80] Gdula R L, Johnson M J A (2006). J Am Chem Soc.

[81] Geyer A M, Wiedner E S, Gary J B, Gdula R L, Kuhlmann N C, Johnson M J A, Dunietz B D, Kampf J W (2008). J Am Chem Soc.

[82] Chiu H-T, Chen Y-P, Chuang S-H, Jen J-S, Lee G-H, Peng S-M (1996). Chem Commun.

[83] Bindl M, Stade R, Heilmann E K, Picot A, Goddard R, Fürstner A (2009). J Am Chem Soc.

[84] Heppekausen J, Stade R, Goddard R, Fürstner A (2010). J Am Chem Soc.

[85] Hickmann V, Alcarazo M, Fürstner A (2010). J Am Chem Soc.

[86] Micoine K, Fürstner A (2010). J Am Chem Soc.

[87] Finke A D, Moore J S (2010). Chem Commun.

[88] Lysenko S, Haberlag B, Daniliuc C G, Jones P G, Tamm M (2011). ChemCatChem.

[89] Fischbach A, Klimpel M G, Widenmeyer M, Herdtweck E, Scherer W, Anwander R (2004). Angew Chem, Int Ed.

[90] Duchateau R (2002). Chem Rev.

[91] Chandrasekhar V, Boomishankar R, Nagendran S (2004). Chem Rev.

[92] Cho H M, Weissman H, Wilson S R, Moore J S (2006). J Am Chem Soc.

[93] Chabanas M, Baudouin A, Copéret C, Basset J M (2001). J Am Chem Soc.

[94] Merle N, Taoufik M, Nayer M, Baudouin A, Le Boux E, Gauvin R M, Lefebvre F, Thivolle-Gazat J, Basset J M (2008). J Organomet Chem.

[95] Bray A, Mortreux A, Petit F, Petit M, Szymanska-Buzar T (1993). J Chem Soc, Chem Commun.

[96] McCullough L G, Listemann M L, Schrock R R, Churchill M R, Ziller J W (1983). J Am Chem Soc.

[97] Suresh C H, Frenking G (2010). Organometallics.

